# Elastography of the Liver in Wilson’s Disease

**DOI:** 10.3390/diagnostics13111898

**Published:** 2023-05-29

**Authors:** Piotr Nehring, Jowita Szeligowska, Adam Przybyłkowski

**Affiliations:** Department of Gastroenterology and Internal Medicine, Medical University of Warsaw, Banacha 1a, 02-097 Warsaw, Poland

**Keywords:** elastography, fibrosis, liver, non-invasive, Wilson’s disease

## Abstract

Staging of liver fibrosis is of special significance in Wilson’s disease as it determines the patient’s prognosis and treatment. Histopathological examination is a standard method for fibrosis assessment; however, non-invasive methods like transient elastography and share wave elastography are believed to be reliable and repetitive and are expected to replace liver biopsy in Wilson’s disease. This article presents a short description of available elastography techniques and the results of the most recent studies on elastography of the liver in patients with Wilson’s disease.

## 1. Introduction

There is a wide spectrum of symptoms of liver dysfunction in Wilson’s disease patients. Although liver disease is frequently insidious, it is estimated that 40–50% of all Wilson’s disease patients present liver symptoms and signs. Moreover, what is unique about Wilson’s disease is that it affects also children, and in this age group, the awareness and reporting of the symptoms are lower than in adults [1]. Liver involvement in Wilson’s disease presents most often with asymptomatic elevation of liver enzymes; however, acute liver failure may be the first manifestation of the disease [2,3]. In the early stages of Wilson’s disease steatosis of the liver is usually reported. Accumulated copper in hepatocytes and consequent mitochondria injury impairs lipid metabolism, which gives a net effect of lipid accumulation in the liver [4,5,6]. However, Wilson’s disease patients usually have normal serum levels of total cholesterol, LDL fraction, and triglycerides [5]. With further progression of the copper overload, liver fibrosis and finally cirrhosis develops.

Staging of liver fibrosis is important for the patient’s prognosis and surveillance. Patients with advanced fibrosis are at risk of clinical complications like ascites, variceal hemorrhage, and encephalopathy [7]. In Wilson’s disease patients who are diagnosed with liver cirrhosis, screening for liver cancer is mandatory, as in cirrhotic patients of other etiology. The liver biopsy is used for Wilson’s disease diagnosis as well as for fibrosis staging, however, histopathological examination of the biopsy specimen enables assessment of only a small part of the liver parenchyma while lesions in the organ are distributed unevenly [8]. Also, the standard imaging techniques of the liver characterize with insufficient sensitivity to precisely assess the degree of fibrosis. For the purpose of non-invasive liver fibrosis evaluation, elastography of the liver and serum markers of fibrosis are widely used.

The newly proposed magnetic resonance elastography (MRE) is based on synchronized MRI acquisition with the measurement of liver elasticity with the use of mechanical (acoustic) waves emitted by an internal device [9,10,11,12]. The contraindications of magnetic resonance elastography are the same as for any MRI scan. The limitations of magnetic resonance elastography are its low availability, high cost, and long duration. It should be considered that Wilson’s disease patients under surveillance require elastography to be repeated many times, therefore the role of MRE may be limited in this group [13].

The presented review aims to summarize and compare available studies on elastographic liver stiffness comparing to clinical categories and/or histopathological reports of the liver in patients with Wilson’s disease.

## 2. Methods

### 2.1. Criteria for Considering Studies for This Review

We searched for randomized controlled trials [RCTs] and/or observational studies with or without a meta-analysis. Included populations were treated children and adults with recently and previously diagnosed Wilson’s disease. We included studies assessing elastography measurements of liver stiffness compared to clinical categories of Wilson’s disease and/or histopathological liver fibrosis advancement. We excluded studies assessing liver stiffness, regardless of the method of measurement, without comparison to clinical categories of Wilson’s disease or liver biopsy histopathological report. Other non-invasive liver injury assessment tools (e.g., APRI, FIB-4) were excluded also, as liver steatosis and liver ions load were not the subjects of this review.

### 2.2. Search Strategy

The PubMed^®^ database (National Library of Medicine, Bethesda, MD, USA) was searched for articles according to the research strategy by two reviewers (P.N. and J.S.). The keywords used in the database searched were ‘Wilson’s disease’, ‘elastography’, and ‘liver’. Due to the limited number of studies, no restrictions by either date or language were used in the search strategies. This review includes research items published and available until February 2023. There was no disagreement between reviewers concerning the eligibility for inclusion. All reviewers agreed on the final set of included articles. PRISMA plot for this review is available as Supplementary Figure S1.

## 3. Results

We have identified 30 research items, and there were no duplicates. However, 21 items were not original studies and, therefore, were excluded from this review. Finally, we have included in this review nine observational studies on patients with Wilson’s disease, four in pediatrics, three in adults, and two in mixed populations. Four of the studies were based on the clinical categorization of liver disease advancement, while the remaining four used liver biopsy results as a comparator to elastography. Due to the heterogeneity of methods and examined populations, there was no premise to perform a meta-analysis. The summary of the results is shown in Table 1.

### 3.1. Studies Evaluating Elastography Alone

The prominent role in liver fibrosis assessment is elastography. Elastography examines the response of tissue to deformation caused by the action of a mechanical or acoustic wave [24]. The lower the elasticity of the tissue, i.e., the greater the stiffness caused by the organ’s fibrosis, the greater the speed of wave propagation, and the greater the test result expressed in kPa (kilopascals) or m/s (meters per second) [25,26,27]. Elastography is a non-invasive, pain-free examination that is easy to repeat [28]. It enables follow-up of patients over a long period, practically lifelong. As it is a convenient and easy method, recommendations are being made for the use of elastography in different groups of patients with liver disease [29]. Elastography techniques are divided into three main types: transient elastography (TE), acoustic radiation force impulse (ARFI) techniques, and strain elastography (SE). Transient elastography uses a mechanical external push. ARFI uses an acoustic internal push. Strain elastography measures tissue deformation caused by pressing the body surface or by internally occurring physiologic motion. In chronic liver disease, transient elastography and AFRI are applicable which use shear-wave imaging (SWI) [30]. ARFI is also called shear-wave elastography (SWE) and is divided into two types: p-SWE and 2D-SWE. Transient elastography is performed with the FibroScan device [31]. Interpretation of the result depends on the etiology of the liver disease [27,32,33]. Moreover, the reliability of the FibroScan results may be affected by significant obesity, ascites, cholestasis, hepatitis, congestive heart failure, and a meal if consumed within 3 h before the test [34,35]. The acoustic radiation force impulse elastography (ARFI) is based on ultrasound imaging in readout B mode [36,37]. Point shear wave elastography (pSWE) is one of the technical approaches for SWI that employs dynamic stress to generate shear waves in parallel or perpendicular dimensions [38,39]. Measurement of the shear wave speed results in qualitative and quantitative estimates of tissue elasticity. Two-dimensional SWE (2D-SWE) is a newer SWI method that uses acoustic radiation force [40,41]. The main factors potentially interfering with measurements are acute and/or severe hepatitis (AST and/or ALT > 5× over normal limit), impaired bile outflow in the liver (obstructive cholestasis), liver congestion, inflammation, congestion, and accumulation of iron, thrombotic material, or fat tissue [19,42,43].

Depending on the study population and liver disease, different cutoff points in kPa for the diagnosis of cirrhosis have been proposed, as suggested by Castera et al. [33] (Table 2). The expert panel of the Society of Radiologists making the current recommendations for elastography suggests the rule of four (5, 9, 13, 17 kPa) for the ARFI techniques for viral etiologies and NAFLD. For alcoholic hepatitis, primary biliary cirrhosis, Wilson disease, autoimmune hepatitis, sclerosing cholangitis, and drug-induced liver disease, there are insufficient data to conclude [32,44]. However, according to the new BAVENO VII guidelines, elastography liver stiffness < 10 kPa in the absence of other clinical or imaging signs is enough to rule out compensated advanced chronic liver disease (cACLD) [45]. According to the same criteria, elastography values between 10 and 15 kPa are suggestive for cACLD, and >15 kPa are highly suggestive for cACLD (Table 3). Following, the BAVENO VII guidelines’ rule of five (10, 15, 20, 25 kPa) for liver stiffness in transient elastography should be used in follow-up to assess the risk of death and decompensation irrespective of the liver disease etiology [45].

Sini et al. compared results of liver stiffness evaluated by transient elastography, serum fibrosis markers, and liver biopsy results in twenty-eight young treated patients with Wilson’s disease using the histopathology-based METAVIR F1-F4 scoring system [46]. The proposed cut-off values of mild (METAVIR F1) and significant (METAVIR F3-4) hepatic fibrosis were 6.6 and 8.4 kPa, respectively. In the study of Sini et al., there was a correlation between METAVIR score and liver stiffness in transient elastography. In this study, there were no significant differences between the mean values of liver stiffness between F1 vs. F2, F2 vs. F3, and F3 vs. F4 subgroups, therefore, cases of mild and no fibrosis could not be differentiated by transient elastography in that study.

In a study by Behairy et al., transient elastography values were compared with the Ishak score in pediatric populations with autoimmune hepatitis, hepatitis C, or Wilson’s disease. There was a correlation between transient elastography results and the Ishak score irrespective of liver disease etiology; however, values of liver stiffness differed between liver disease etiologies [17]. The mean liver stiffness was highest in patients with AIH and with Wilson’s disease, and lowest in HCV, 22.13 kPa, 20.05 kPa, and 5.75 kPa, respectively. In this study, all patients with Wilson’s disease had fibrosis category two or higher in Ishak score, 30% had moderate fibrosis (Ishak score two to three), and 70% had severe fibrosis (Ishak score four to six). In the Behairy et al. study, liver stiffness in elastography correlated with liver fibrosis reflected with semi-quantitative Ishak score and linear percentage of fibrosis area fraction in Wilson’s disease, AIH, and HCV cirrhosis [17].

In a study by Stefanescu et al., in nine children with Wilson’s disease, liver stiffness measured with transient elastography was the highest at the time of diagnosis and decreased during treatment in parallel with an increase in urinary copper concentration [19]. In this study, two out of nine patients were diagnosed with cirrhosis. All patients were treated with zinc or penicillamine. The median liver stiffness decreased during treatment from a mean of 15.1 kPa to 6.1 kPa during the 24-month follow-up. Based on these findings, repeated liver stiffness measurements may be useful in assessing the progression/regression of liver fibrosis in Wilson’s disease. Data from Stefanescu et al. suggest that liver stiffness in patients with Wilson’s disease decreases during copper-lowering therapy. This finding supports the previous hypothesis that high baseline liver stiffness values may be a consequence of intrahepatic copper deposition. Preliminary data of Stefanescu et al. are limited with a lack of histopathological copper liver deposition and fibrosis assessment, which require confirmation in biopsy-based vs. elastography studies.

On the contrary, in the study by Przybyłkowski et al. on adults, transient elastography and share wave elastography did not correlate with the Ishak score or collagen proportionate area in liver biopsy in the study of sixty adult patients with Wilson’s disease [20]. In this study, all patients were on treatment, either with zinc, d-penicillamine, or bis-choline tetra thiomolybdate. The study by Przybyłkowski et al. included patients with Ishak scores ranging from one to five, without patients with a score of six representing cirrhosis. Forty-eight patients (80%) had an Ishak score of three or lower, and cirrhosis (Ishak score ≥ 5) was diagnosed in five patients (8%). In this study, there was a tendency for higher liver stiffness in Wilson’s disease patients with Ishak scores of one to two and a drop in Ishak scores from three to five. The highest liver stiffness of 8.5 kPa in this study was in patients with an Ishak score of three; however, the median duration of treatment was eight years which may bias ultrasonographic findings due to heterogeneity of copper accumulation in patients treated for many years compared to those newly diagnosed with Wilson’s disease [20]. The researchers in the Przybyłkowski et al. study concluded that the lack of linear correlation of elastography with histological assessment of liver fibrosis in Wilson’s disease may be a consequence of the varying liver involvement patterns in different stages of the disease. The histopathologic findings in Wilson’s disease comprise steatosis, glycogenation of nuclei, mitochondrial changes, portal and periportal inflammation, parenchymal necrosis, bridging fibrosis, intracytoplasmic eosinophilic Mallory bodies, micronodular or mixed micro– and macronodular cirrhosis, cavitation, deposition of copper and lipogranulomas [47]. It was previously shown that liver biopsy does not correlate with clinical parameters or initial presentation in Wilson’s disease patients [16].

A recently published study by Yavuz et al. of the pediatric population aimed to demonstrate parenchymal changes in the liver and pancreas related to copper accumulation using ultrasound and assess the effectiveness of two-dimensional shear wave elastography in Wilson’s disease [21]. In this study, 2D-share wave elastography showed a significant increase in liver stiffness in 25 children with Wilson’s disease compared to 37 healthy controls. Moreover, in the Yavuz et al. study, Wilson’s disease follow-up duration showed to be significantly associated and moderately correlated with liver tissue stiffness [21]. The Yavuz et al. study included patients with clinical categories I, II, and III, which comprise patients without clinical signs of liver cirrhosis and cirrhosis decompensation. Most of the included patients were in category II, only with biochemical abnormalities, there were no patients with liver cirrhosis involved. The lack of histological assessment of liver fibrosis advancement is a limitation of the study. However, the presented results are in line with most of the previous studies.

### 3.2. Studies Evaluating Elastography and Serological Markers

Several serum markers of liver fibrosis are used in non-invasive assessment of liver involvement. Direct markers of liver fibrosis are products of extracellular matrix synthesis or destruction [48] and are closely related to fibrogenesis. The direct markers include laminin, hyaluronic acid, YKL-40 glycoprotein, N-terminal propeptide of procollagen type III (PIIINP), C-terminal pro-peptide of procollagen type I (PICP), TGF-β, and matrix metalloproteinases (MMP) [49]. Indirect markers of liver fibrosis, which are calculated with mathematical algorithms, are far more commonly used in practice than direct serum markers [50]. The Fibrosis-4 (AST, ALT, PLT, and patient age; FIB-4) and AST to Platelet Ratio Index (APRI), as well as FibroTest (GGT, bilirubin, alpha-2-macroglobulin, haptoglobin, apolipoprotein A-I), have been validated in chronic liver diseases: alcoholic liver disease, NAFLD, chronic hepatitis C, and chronic hepatitis B [51,52]. The FIB-4 and APRI were found useful also for patients with Wilson’s disease [14,18]. In a study by Paternostro et al. with one hundred eighty Wilson’s disease patients, APRI and FIB-4 were significantly higher in patients with liver cirrhosis compared to patients without liver cirrhosis. The proposed cut-off values to exclude liver cirrhosis in patients with Wilson’s disease were <1.5 for APRI and <3.25 for FIB-4 with a specificity of 93% and 95%, respectively [18]. The fibrosis probability index (FPI) and the HepaScore FibroMeter algorithm BARD score (BMI, AST/ALT ratio), despite gaining acceptance by clinicians, have not yet been assessed in Wilson’s disease [32,46,51,52,53,54]. In the study by Karlas et al. of fifty adults with Wilson’s disease, transient elastography showed an increase in liver tissue stiffness in different stages of hepatic manifestation. Based on the results of Karlas et al., it may be possible to discriminate liver cirrhosis in Wilson’s disease patients (categories IV and V) at a cut-off value of 6.1 kPa [14]. The patients with fibrosis had lower liver stiffness compared to patients with liver cirrhosis, 7.0 kPa, and 10.1 kPa, respectively. The elastographic liver stiffness values correlated not only with the clinical categories of Wilson’s disease, but also with other non-invasive biochemically based indices (APRI, FIB-4, and Forns). Therefore, Karlas et al. proposed the clinical classification of five categories of Wilson’s disease. In clinical category I, there are no laboratory, ultrasound, or clinical abnormalities. In clinical category II, only biochemical abnormalities are found. In clinical category III, characteristic morphological abnormalities may be found on an ultrasound of the liver (heterogeneous or increased parenchymal echogenicity without signs of liver cirrhosis). Clinical category IV describes compensated liver cirrhosis stadium, whereas clinical category V describes liver cirrhosis decompensation.

A Hwang et al. study involved fifty-five patients with Wilson’s disease. The increased liver stiffness in parallel with the clinical stage of hepatic manifestation was demonstrated using TE and 2D-SWE [15]. The study by Hwang et al. and the Karlas et al. studies do not include a histopathological quantitative assessment of liver fibrosis and liver copper deposits.

A study by Paternostro et al. included one hundred-eight adult patients with Wilson’s disease and aimed to evaluate transient elastography and laboratory-based non-invasive markers for fibrosis assessment [18]. In this study, forty-four patients had newly diagnosed Wilson’s disease i.e., less than one year before the evaluation, and 144 patients were diagnosed over one year before evaluation (previously diagnosed). The study included patients treated with various drugs (d-penicillamine, trientine, zinc, bis-choline tetra thiomolybdate, or combinations). Liver cirrhosis was found in 51 patients: 27.1% (14 recently diagnosed and 37 previously diagnosed with Wilson’s disease). Fibrosis stages to three in Ishak score was found in 59 patients: 31.4% (12 recently diagnosed and 47 previously diagnosed with Wilson’s disease). Median liver stiffness in recently diagnosed Wilson disease patients was 8.8 kPa, whereas in previously diagnosed 6.3 kPa. In the area under the curve (AUC), the analysis for diagnosing cirrhosis by transient elastography optimal cutoff was ≥9.9 kPa with a sensitivity of 61% and specificity of 85%. In recently diagnosed patients with Wilson’s disease, this cut-off had 100% sensitivity and 83% specificity, whereas in previously diagnosed, 46% sensitivity and 86% specificity. The authors concluded that transient elastography and non-invasive fibrosis scores are useful to identify cirrhosis in patients with recently diagnosed Wilson’s disease and proposed a new cutoff value of liver stiffness for cirrhosis ≥ 9.9 kPa [14].

For the assessment of liver steatosis, the attenuated coefficient (ATT) had been recently used. ATT is a non-invasive and quantitative liver fat measurement method based on ultrasound B mode presentation [55]. The estimation of the fat volume is performed according to the difference in the degree of attenuation of the signal received by the ultrasound probe. ATT has been developed 20 years ago and since then extensively studied in NAFLD [56]; however, the first study on ATT in Wilson’s disease has been published recently [22].

Recently, a Wang et al. study based on a combination of non-invasive methods including elastography and ATT in Wilson’s disease has been published. ATT in combination with APRI, FIB-4, and elastographically measured liver stiffness have been proven to be accurate for the diagnosis of hepatic steatosis in patients with Wilson’s disease [22]. In a Wang et al. study with sixty-two children and adolescents with Wilson’s disease, ATT, elastographic liver stiffness, APRI, and FIB-4 were compared between groups of patients categorized for liver involvement by collagen type IV (CIV), hyaluronic acid (HA), laminin (LN), and pre-collagen type III N-terminal peptide (PIIINP) serum concentration. Group One had normal CIV, HA, LN, and PIIINP; group two had an elevation of one or two markers; whereas group three had an elevation of three markers out of four. In this study, the sensitivity for the diagnosis of hepatic steatosis in Wilson’s disease was 89.47% with the cut-off value of ATT of 0.73 dB/cm/MHz, while the combination of ATT with APRI and FIB-4, or the combination of ATT with elastography, APRI, and FIB-4, showed a better diagnostic accuracy [22]. In the same study, elastographically measured liver stiffness was increased in all groups. Among 62 children with Wilson’s disease in the Wang et al. study, 54 presented liver involvement. However, the study lacks histopathological liver fibrosis assessment or reference to Wilson’s disease clinical categories proposed by Karlas et al.

Aforementioned studies on liver stiffness combined with serum markers of fibrosis lack information on how the precision of the assessment of liver fibrosis changes with the use of a combination of methods. Moreover, in most cases these are pediatric population studies where liver biopsies have not been performed, while this is the reference liver fibrosis assessment method.

The number of published studies on the use of elastography in Wilson’s disease is limited, mainly due to the small size of the population of people diagnosed with Wilsons’s disease. Most published studies treat the pediatric and adult populations separately. In addition, the studies differ in methodology by comparing liver elastographic stiffness with clinical stages of Wilson’s disease or liver fibrosis results obtained by biopsy. All currently available studies in the pediatric and adult population were presented in this review, both those comparing liver elastographic stiffness with clinical categories of Wilson’s disease and hepatic fibrosis as assessed by liver biopsy. It is worth mentioning two factors important in the construction of studies on liver fibrosis in Wilson’s disease: these are the assessments of the accumulation of divalent ions in the liver parenchyma (e.g., copper and iron) at the time of treatment of Wilson’s disease. The elastographic stiffness of the liver may be related to the accumulation of copper in the liver. On the other hand, copper chelating therapy may reduce elastographic liver stiffness independent of liver fibrosis.

## 4. Conclusions

The share wave or transient elastography combined with cyclic imaging examinations of the liver seems to be the optimal and sufficient procedure in the assessment of liver morphology in Wilson’s disease, both in pediatric and adult populations. Based on the available results, transient elastography and share wave elastography seem to be comparably accurate tools. There is a slight difference in the results of children and adults, those recently diagnosed, and patients treated for many years. The differences, together with the heterogeneity of liver injury and progressive copper accumulation, comprise a heterogenous image in Wilson’s disease. Thus, expert agreement on liver stiffness cutoff values for particular stages of fibrosis in Wilson’s disease population is avidly expected.

In light of the available results, it can be concluded that a single measurement of liver stiffness is not sufficient to accurately stage fibrosis in Wilson’s disease, although it allows us to forge or confirm fibrosis. Repeated elastography in the same patient, however, allow for a more precise assessment of disease progression or regression. The combination of several methods of non-invasive assessment of fibrosis increases the chance of a more precise assessment.

Nevertheless, a non-invasive liver stiffness measure is on the way to replace liver biopsy in Wilson’s disease.

## Figures and Tables

**Table 1:** A short comparison of studies assessing elastography in liver fibrosis in Wilson’s disease population.

Study	Population	Clinical Category	Histopathological Advancement	* TE, kPa	* 2D SWE, kPa
Karlas et al. (2012) [14]	50 adults	III IV–V	-	7.0 ± 2.2 10.1 ± 6.73	-
Hwang et al. (2020) [15]	55 children and young adults	I II III IV	-	4.10 (1.45) 5.10 (1.80) 8.80 (7.30) 17.30 (1.90)	4.60 (1.15) 6.40 (2.70) 7.80 (5.75) 15.00 (2.65)
Sini et al. (2013) [16]	35 adults	-	METAVIR F1 METAVIR F2 METAVIR F3 METAVIR F4	5.6 (3.8–8.1) 7.35 (5.6–17.1) 10.4 (6.7–16.8) 13.5 (8.7–18.4)	-
Bahairy et al. (2016) [17]	20 children	-	ISHAK 2 ISHAK 3 ISHAK 4 ISHAK 5 ISHAK 6	6.00 8.30 ± 0.84 15.75 ± 0.96 28.49 ± 4.93 30.33 ± 9.87	-
Paternostro et al. (2020) [18]	88 adults	-	ISHAK 4–6	Cut-off ≥ 9.9	-
Stefanescu et al. (2016) [19]	9 children	-	-	15.1 (5.1–66)	
Przybyłkowski et al. (2021) [20]	60 adults	-	ISHAK 1 ISHAK 2 ISHAK 3 ISHAK 4 ISHAK 5	6.6 (3.4) 7.2 (3.4) 6.2 (3.8) 5.5 (2.8) 5.0 (3,1)	7.8 (1.9) 8.3 (1.5) 8.5 (4.1) 7.9 (2.1) 6.7 (21.1)
Yavuz et al. (2022) [21]	25 children and young adults	I–III	-	1.06 (0.91–1.32)	1.06 (0.91–1.32) [m/s] **
Wang et al. (2023) [22]	62 children	-	normal liver steatosis	-	1.47 (0.45) [m/s] ** 1.50 (0.56) to 1.57 (0.82)

* Mean ± SD or median (IQR or range); ** data available as [m/s]. Clinical category as described in Karlas et al. study [14]; ISHAK score as defined in [23].

**Table 2:** The original METAVIR cutoff values in the assessment of liver fibrosis and cirrhosis based on elastographic liver stiffness in kPa [33].

Stage	Elastographic Liver Stiffness	Interpretation
METAVIR F0	<2.5 kPa	Absence of fibrosis
METAVIR F1	2.5–7.0 kPa	Mild fibrosis
METAVIR F2	7.0–9.5 kPa	Moderate fibrosis
METAVIR F3	9.5–12.5 kPa	Severe fibrosis
METAVIR F4	>12.5 kPa	Liver cirrhosis

METAVIR—Meta-analysis of Histological Data in Viral Hepatitis score.

**Table 3:** Recommendation for interpretation of liver stiffness values obtained via elastography according to BAVENO VII guidelines [45].

Liver Stiffness Value	Recommendation
<10 kPa	In the absence of other clinical or imaging signs is enough to rule out the cACLD
10–15 kPa	Suggestive for cACLD
>15 kPa	Highly suggestive for cACLD

cACLD—compensated advanced chronic liver disease.

## Data Availability

No additional data are available.

## Supplementary Materials

The following supporting information can be downloaded at: https://www.mdpi.com/article/10.3390/diagnostics13111898/s1; Figure S1: Role of elastographic liver fibrosis assessment in Wilson’s disease (PRISMA) [57].
