# Supplementary material for: Elastography of the Liver in Wilson’s Disease
Source: Diagnostics (Basel). 2023 May 29;13(11):1898. doi: 10.3390/diagnostics13111898 (PMC10253198; doi:10.3390/diagnostics13111898)
Supplement: Supplementary file 1 [file diagnostics-13-01898-s001.zip › diagnostics-2370396-supplementary.pdf]

Figure S1 Role of elastographic liver fibrosis assessment in Wilson’s disease (PRISMA).

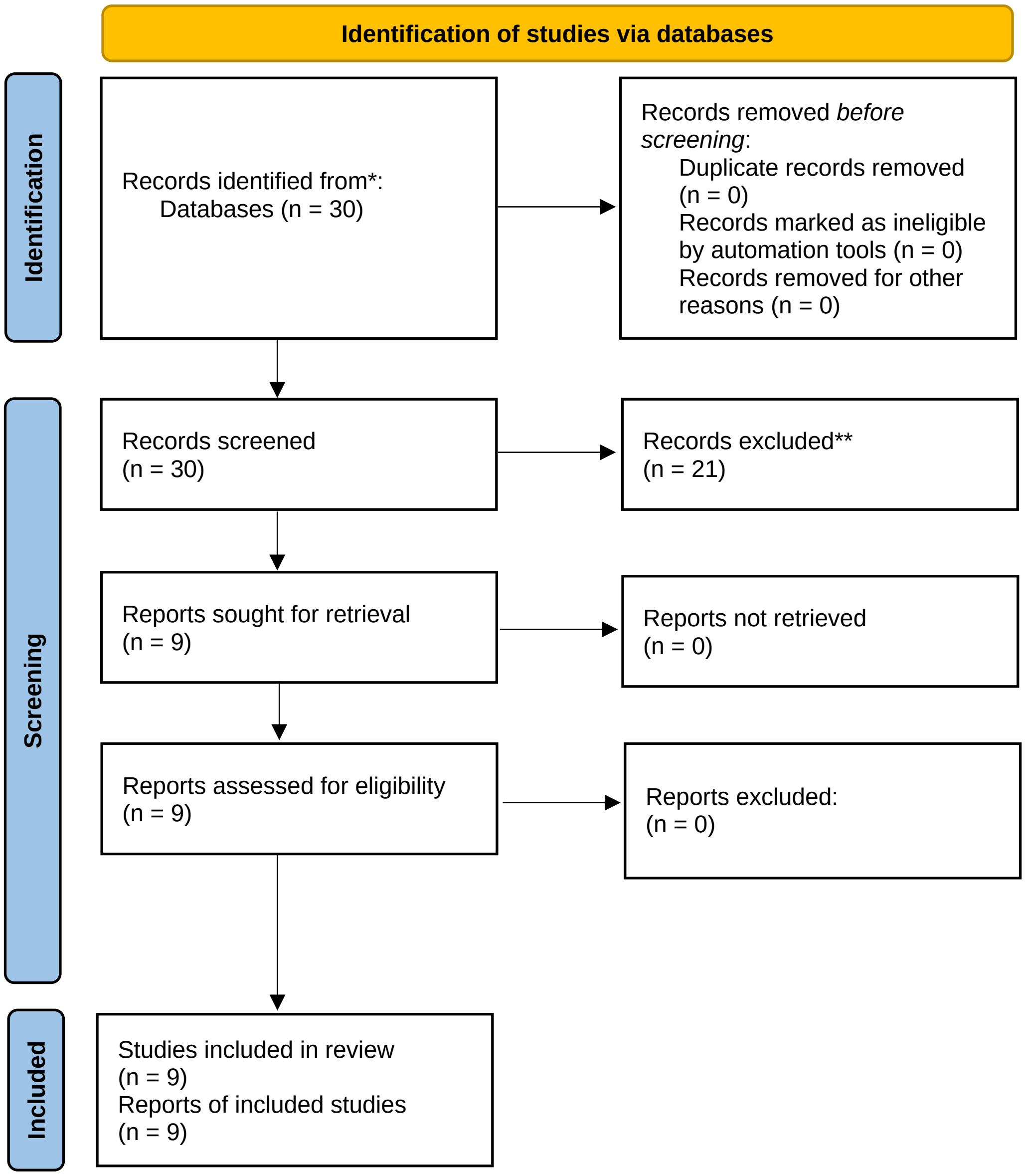

*Consider, if feasible to do so, reporting the number of records identified from each database or register searched (rather than the total number across all databases/registers).

**If automation tools were used, indicate how many records were excluded by a human and how many were excluded by automation tools.

From: Page MJ, McKenzie JE, Bossuyt PM, Boutron I, Hoffmann TC, Mulrow CD, et al. The PRISMA 2020 statement: an updated guideline for reporting systematic reviews. BMJ 2021;372:n71. doi: 10.1136/bmj.n71

For more information, visit: http://www.prisma-statement.org/
